# Attitudes of Lebanese adults regarding COVID-19 vaccination

**DOI:** 10.1186/s12889-021-10902-w

**Published:** 2021-05-27

**Authors:** Carina Kasrine Al Halabi, Sahar Obeid, Hala Sacre, Marwan Akel, Rabih Hallit, Pascale Salameh, Souheil Hallit

**Affiliations:** 1 Faculty of Medicine and Medical Sciences, Holy Spirit University of Kaslik (USEK), Jounieh, Lebanon; 2 Faculty of Arts and Sciences, Holy Spirit University of Kaslik (USEK), Jounieh, Lebanon; 3 INSPECT-LB (Institut National de Santé Publique, d’Épidémiologie Clinique et de Toxicologie-Liban), Beirut, Lebanon; 4 Research and Psychology Departments, Psychiatric Hospital of the Cross, Jal Eddib, Lebanon; 5 School of Pharmacy, Lebanese International University, Beirut, Lebanon; 6 Infectious Disease Department, Bellevue Medical Center, Mansourieh, Lebanon; 7 Infectious Disease Department, Notre-Dame des Secours University Hospital, Byblos, Cyprus; 8 Faculty of Pharmacy, Lebanese University, Hadat, Lebanon; 9 University of Nicosia Medical School, Nicosia, Cyprus

**Keywords:** Covid-19, Vaccine, Hesitancy, Lebanon, Pandemic

## Abstract

**Background:**

COVID-19 was first detected in Lebanon on February 21, 2020; it reached its peak in January 2021, with a total number of 418,448 confirmed cases and 5380 deaths (until March 15, 2021). Gaining insight into factors regarding willingness or refusal for vaccination might guide our goals in raising the awareness and target efforts to increase acceptance of the COVID-19 vaccine and maximize the uptake. Therefore, this study aims to assess the intent to receive the COVID-19 vaccine among Lebanese adults and the factors associated with vaccine refusal.

**Methods:**

We conducted a cross-sectional study during November–December 2020 among Lebanese adults from all Lebanese regions using a survey tool with closed-ended questions that included sociodemographic data and questions about vaccine hesitancy, knowledge, attitude, practice, and fear of COVID-19. We used the snowball technique to collect the data because of the COVID-19 imposed lockdown.

**Results:**

Of the 579 participants, 21.4% were willing to receive the vaccine, 40.9% refused, and the remainder were unsure of their response. More vaccine hesitancy (adjusted odds ratio (aOR) = 1.06; 95% CI 1.03–1.09) was significantly associated with more odds of disagreeing/ strongly disagreeing on receiving the COVID-19 vaccine compared to being neutral. More vaccine hesitancy (aOR = 0.95; 95% CI 0.91–0.99), female gender compared to males (aOR = 0.53; 95% CI 0.32–0.87), and being married compared to single (aOR = 0.53; 95% CI 0.29–0.98) were significantly associated with lower odds of agreeing/strongly agreeing on receiving the COVID-19 vaccine compared to being neutral.

**Conclusion:**

Overall, our findings revealed a high percentage of people (40%) who strongly disagreed with receiving the vaccine, mainly females, married participants, and those who have a general vaccine hesitancy. Moreover, no significant association was found with knowledge, attitude, or prevention practice regarding COVID-19. Targeted efforts are necessary to increase acceptance of a COVID-19 vaccine among the Lebanese population to control the COVID-19 pandemic. Further studies with a larger sample size are warranted to validate our results and provide better insights into the underlying reasons for refusing vaccination.

## Background

The coronavirus disease 19 (COVID-19), caused by novel severe acute respiratory syndrome corona-virus 2 (SARS-CoV-2), is responsible for the worst pandemic ever and has contributed to health, lives, and economic losses [1, 2]. It has emerged in Wuhan, China at the end of December 2019 and rapidly spread globally, leading to nearly 2 million deaths and 98 million confirmed cases till January 23, 2021 [3].

Lebanon reported its first case on February 21, 2020, a Lebanese woman returning Lebanon from Iran [4]. Two other cases were suspected and followed after being quarantined in Beirut hospital [5]. By March 15, 2020, the government declared a total lockdown for two weeks. Series of stern measures were adopted then, including restrictions on vehicular movements as per the odd/even plate numbers alongside daily curfew were extended until June and July 2020, respectively [6, 7]. These restrictions have contributed to slowing the increase in patient numbers for a few months. As cases spiked again, Lebanon entered another lockdown in November 2020 [8], followed by another in early 2021 [9]. As of March 15, 2021, Lebanon counts up to 418,448 confirmed cases and 5380 deaths [3].

A study [10] has revealed that people with positive beliefs and attitudes about COVID-19 vaccination tend to be vaccinated when a vaccine becomes available. It has shown that 64% of participants who reported they were very likely to be vaccinated were those who had a lower belief that vaccination would be unsafe, were more familiar with the disease and vaccination, were older, and had been vaccinated against the flu in the winter [10]. Other studies showed that COVID-19 vaccine rejection is strongly correlated with mistrust of its benefit, worry about unforeseen future effects, preferences for natural immunity, and hesitancy for taking any type of vaccine [11]. The latter was associated with personal experiences with vaccinations, barriers to access, alternative belief models, limited knowledge, and profound misunderstanding about how vaccines work [11].

Gaining insight into factors regarding willingness or refusal for vaccination might guide our goals in raising the awareness and target efforts to increase acceptance of the COVID-19 vaccine and maximize the uptake. Therefore, the study objective is to assess the intent to receive the COVID-19 vaccine among Lebanese adults and the factors associated with vaccine refusal.

## Methods

### Study design

We conducted a cross-sectional study between November and December 2020, during the COVID-19 imposed lockdown, when vaccination was actively discussed, and the Lebanese government announced it would be available in Lebanon within the coming months. We used the snowball technique to select the sample from the five governorates of Lebanon (Beirut, Beqaa, Mount Lebanon, South Lebanon, and North Lebanon). The first page of the questionnaire included an explanation of the study topic and objective and a statement ensuring the anonymity of respondents. People above 18 years old and living in Lebanon were eligible to participate. All methods were performed in accordance with the relevant guidelines and regulations.

### Sample size calculation

According to the G-power software, and based on an effect size f2 = 2%, an alpha error of 5%, a power of 80%, and taking into consideration 16 factors to be entered in the multivariable analysis, the minimum required sample was 395.

### Questionnaire

The questionnaire was self-administered and in Arabic, the native language in Lebanon. It consisted mainly of closed-ended questions covering sociodemographic features, knowledge, attitude, and practice, in addition to a scale-based section about different factors.

#### Sociodemographic data and general questions

This section of the questionnaire collected sociodemographic data of the participants, including age, educational level, income, marital status, and anthropometric measurements (height and weight). It also included questions about the history of medical illnesses, the health status of people living with the participant, willingness to take the COVID-19 vaccine, the source of information about COVID-19, having tested positive for COVID-19, believing coronavirus existed, and following the recommendations of the Ministry of Public Health.

#### Vaccine hesitancy questions

This section was developed from previous scales and published data about the aspects of vaccine hesitancy. It included questions from two tools currently available for assessing vaccine hesitancy: a scoping review protocol [12] and the vaccine hesitancy scale: psychometric properties and validation [13]. The first tool consists of 17 items rated on a 4-point Likert scale from strongly agree (1) to strongly disagree (4); the second is a 9-item scale with three modalities: yes, no, and I do not know.

#### Knowledge, attitude, and practice about COVID-19

This part included questions about knowledge (26 items), attitude (19 items), and practice (12 items) selected from a previous study [14]. All items were rated on a 5-point Likert scale from never (0) to always (4).

#### Scale-based category: the fear of COVID-19 scale

Since the COVID-19 pandemic can worsen psychological health and exacerbate social isolation, which is strongly associated with increased anxiety and depression, the fear of COVID-19 scale (FCV-19S) was used to assess fear of COVID-19 among participants. This 7-item tool is scored on a 5-point Likert scale from 1 (strongly disagree) to 5 (strongly agree). The total score, ranging from 7 to 35, is calculated by summing all responses. Higher scores indicate greater levels of fear of COVID-19 [15].

### Translation procedure

A clinical psychologist performed the forward translation from English into Arabic. A professional medical writer verified this translation. The backward translation was performed by a second clinical psychologist, unaware of the scales’ notions and fluent in Arabic. The principal investigator matched the back-translated English questionnaire with the original one to detect inconsistencies and solve discrepancies between the two versions.

### Statistical analysis

Statistical Package for the Social Sciences (SPSS) 25 was used for the data analysis. Since the data was collected via a link, no missing values were recorded as all questions were required. The Chi-square test was used to compare categorical variables, whereas the ANOVA test was used to compare three means. Multinomial logistic regression was conducted, taking the willingness to receive the COVID-19 vaccine as the dependent variable. All variables that showed a *p* < 0.2 were taken as independent variables in the final model. Significance was set at a *p* < 0.05.

## Results

The Cronbach’s alpha values were as follows: vaccine hesitancy (0.841), knowledge (0.896), attitude (0.828), practice (0.886), and FCV-19S (0.874).

### Sociodemographic and other characteristics of the participants

The total number of participants was 579, with a mean age of 24.94 ± 9.45 years and 76.2% females. Also, 40.9% were unwilling to receive the COVID-19 vaccine, 37.7% were neutral, whereas 21.4% were in favor of the vaccine. Other characteristics are summarized in Table 1.

**Table 1:** Characteristics of survey participants on COVID-19 vaccine hesitancy in Lebanon, November through December 2020 (*N* = 579)

Variable	N (%)
**Gender**	
Male	138 (23.8%)
Female	441 (76.2%)
**Marital status**	
Single/ widowed/ divorced	446 (77.0%)
Married	133 (23.0%)
**Education**	
Complementary or less	35 (6.0%)
Secondary	84 (14.5%)
University	460 (79.4%)
**Living with a person at risk (pregnant, cardiovascular disease, respiratory disease, patients with cancer, immunocompromised)**	
No	204 (35.2%)
Yes	375 (64.8%)
**Being a person at risk (pregnant, cardiovascular disease, respiratory disease, patients with cancer, immunocompromised)**	
No	401 (69.3%)
Yes	178 (30.7%)
**Willingness to do the COVID-19 vaccine**	
Strongly disagree/ Disagree	237 (40.9%)
Neutral	218 (37.7%)
Agree/ Strongly agree	124 (21.4%)
	**Mean ± SD**
**Age (in years)**	24.94 ± 9.45
**Household crowding index**	1.10 ± 0.45
**Vaccine hesitancy**	32.01 ± 6.48
**Knowledge**	19.18 ± 5.70
**Attitude**	13.14 ± 3.59
**Practice**	7.78 ± 3.35
**Fear of COVID-19**	17.02 ± 5.62

### Bivariate analysis

A significantly higher percentage of males and single participants agreed to receive the COVID-19 vaccine. A higher mean vaccine hesitancy was found in those who disagreed/strongly disagreed on receiving the COVID-19 vaccine compared to those who were neutral or who agreed/strongly agreed on receiving it. No significant association was found between the willingness to receive the COVID-19 vaccine and the following variables: education level, living with a person at risk of contracting COVID-19, being a person at risk of contracting COVID-19, having received the flu vaccine, having been previously infected with COVID-19, knowing a family member/friend who has previously contracted COVID-19, anxiety that someone close catches COVID-19, believing that coronavirus is a hoax, age, household crowding index, knowledge and attitude towards COVID-19, and fear of COVID-19 (Table 2).

**Table 2:** Bivariate analysis of variables associated with the willingness to do the COVID-19 vaccine

Variable	Willingness to do the COVID-19 vaccination			***p***
	Strongly disagree/ disagree	Neutral	Agree/ Strongly agree	
**Gender**				**0.006**
Male	48 (34.8%)	47 (34.1%)	43 (31.2%)	
Female	189 (42.9%)	171 (38.8%)	81 (18.4%)	
**Marital status**				**0.04**
Single/ widowed/ divorced	178 (39.9%)	162 (36.3%)	106 (23.8%)	
Married	59 (44.4%)	56 (42.1%)	18 (13.5%)	
**Education**				0.391
Complementary or less	15 (42.9%)	12 (34.3%)	8 (22.9%)	
Secondary	42 (50.0%)	29 (34.5%)	13 (15.5%)	
University	180 (39.1%)	177 (38.5%)	103 (22.4%)	
**Living with a person at risk (pregnant, cardiovascular disease, respiratory disease, patients with cancer, immunocompromised)**				0.180
No	89 (43.6%)	80 (39.2%)	35 (17.2%)	
Yes	148 (39.5%)	138 (36.8%)	89 (23.7%)	
**Being a person at risk (pregnant, cardiovascular disease, respiratory disease, patients with cancer, immunocompromised)**				0.286
No	156 (38.9%)	154 (38.4%)	91 (22.7%)	
Yes	81 (45.5%)	64 (36.0%)	33 (18.5%)	
**Flu vaccine this year**				0.909
No	197 (41.0%)	179 (37.3%)	104 (21.7%)	
Yes	40 (40.4%)	39 (39.4%)	20 (20.2%)	
**Participant diagnosed with coronavirus infection**				0.319
No	187 (41.5%)	163 (36.1%)	101 (22.4%)	
Yes	50 (39.1%)	55 (43.0%)	23 (18.0%)	
**Family member/friend diagnosed with coronavirus infection**				0.260
No	116 (44.3%)	90 (34.4%)	56 (21.4%)	
Yes	121 (38.2%)	128 (40.4%)	68 (21.5%)	
**Anxiety that someone close catches coronavirus**				0.625
No	69 (39.4%)	71 (40.6%)	35 (20.0%)	
Yes	168 (41.6%)	147 (36.4%)	89 (22.0%)	
**Thought that coronavirus is a hoax**				0.298
No	203 (40.7%)	184 (36.9%)	112 (22.4%)	
Yes	34 (42.5%)	34 (42.5%)	12 (15.0%)	
**Age**	24.44 ± 8.13	25.82 ± 10.78	24.35 ± 9.27	0.223
**Household crowding index**	1.12 ± 0.46	1.07 ± 0.40	1.12 ± 0.51	0.392
**Vaccine hesitancy**	41.25 ± 6.42	43.47 ± 6.13	45.46 ± 6.27	**< 0.001**
**Knowledge**	19.03 ± 5.95	19.18 ± 5.81	19.47 ± 5.01	0.787
**Attitude**	12.89 ± 3.73	13.21 ± 3.36	13.48 ± 3.68	0.306
**Fear of COVID-19**	17.20 ± 6.02	17.19 ± 5.48	16.38 ± 5.05	0.359

Numbers in bold indicate significant *p*-values; post hoc analysis: vaccine hesitancy and willingness to do the COVID vaccine: strongly disagree/disagree vs neutral *p* = 0.001; neutral vs agree/strongly disagree *p* < 0.001

### Multivariable analysis

More vaccine hesitancy (adjusted odds ratio (aOR) = 1.06; 95% CI 1.03–1.09) was significantly associated with more odds of disagreeing/strongly disagreeing on receiving the COVID-19 vaccine compared to being neutral (Table 3, Model 1).

**Table 3:** Multivariable analysis: Multinomial regression taking the willingness to do the COVID-19 vaccine

Variable	***p***	aOR	95% Confidence Interval	
Model 1: Willingness to do the COVID-19 vaccine (strongly disagree/disagree vs neutral* category)				
Vaccine hesitancy	**0.001**	1.06	1.03	1.09
Knowledge score	0.389	1.02	0.98	1.06
Attitude score	0.524	0.98	0.92	1.05
Fear of COVID-19 score	0.914	1.002	0.97	1.04
Gender (females vs males*)	0.676	1.10	0.70	1.75
Marital status (married vs single*)	0.596	0.89	0.57	1.38
Living with a person at risk (yes vs no*)	0.629	0.90	0.60	1.37
Being a person at risk (yes vs no*)	0.530	1.15	0.74	1.78
Model 2: Willingness to do the COVID-19 vaccine (agree/strongly agree vs neutral* category)				
Vaccine hesitancy	**0.01**	0.95	0.91	0.99
Knowledge score	0.708	0.99	0.94	1.04
Attitude score	0.44	1.03	0.95	1.12
Fear of COVID-19 score	0.225	0.98	0.94	1.02
Gender (females vs males*)	**0.013**	0.53	0.32	0.87
Marital status (married vs single*)	**0.041**	0.53	0.29	0.98
Living with a person at risk (yes vs no*)	0.157	1.45	0.87	2.43
Being a person at risk (yes vs no*)	0.864	0.95	0.56	1.64

*Reference group; numbers in bold indicate significant *p*-values; *aOR* adjusted odds ratio Goodness of fit Pearson value = 1175.82; *p* = 0.001; Pseudo *R*2 = 11.2%

More vaccine hesitancy (aOR = 0.95; 95% CI 0.91–0.99), female gender compared to males (aOR = 0.53; 95% CI 0.32–0.87), and being married compared to single (aOR = 0.53; 95% CI 0.29–0.98) were significantly associated with lower odds of agreeing/strongly agreeing on receiving the COVID-19 vaccine compared to being neutral (Table 3, Model 2).

### Association between the practice score and the willingness to do the COVID-19 vaccine

**Fig. 1:**
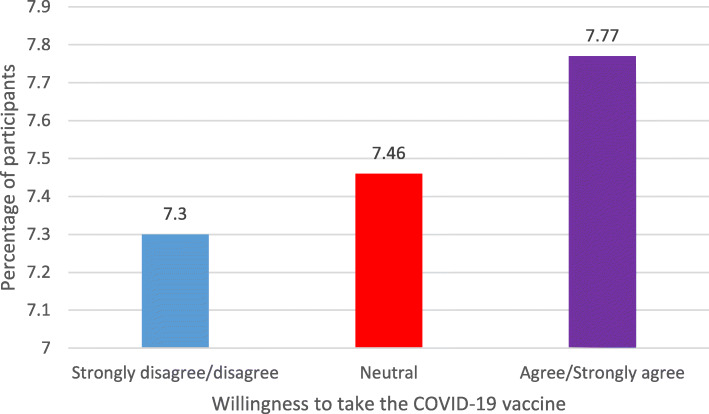
Association between willingness to do the COVID-19 vaccine and practice after adjustment over potential confounding variables (age, gender, household crowding index, education level)

No significant association was found between the willingness to receive the COVID-19 vaccine and the practice score F (2,576) = 0.657; *p* = 0.519 (Fig. 1). No significant difference was found when the categories were compared two by two.

## Discussion

Our study revealed that less than a quarter of Lebanese adults expressed willingness to accept the COVID-19 vaccine when it becomes available, a rate far below those of the UK (64%) [10] and the US (57.6%) [16]. This discrepancy between Lebanon’s figures and those of other countries should prompt the Lebanese government to raise awareness about the importance of vaccination while ensuring equitable vaccine distribution.

Vaccine hesitancy was significantly associated with higher odds of disagreeing with receiving the COVID-19 vaccine, in line with findings from France, the US, and Greece, where vaccine hesitancy is also associated with decreased COVID-19 vaccine uptake. Moreover, previous studies had shown that social and educational backgrounds and complex information delivered through the media might have played a role in more hesitancy toward vaccination [17, 18], making people more fearful of vaccine side effects, particularly when some local media reported multiple deaths in several countries of people who received the COVID-19 vaccine. An American study [16] has also demonstrated that mistrust and limited knowledge about the vaccine contributed to increased vaccine hesitancy. Moreover, some people do not believe in the effectiveness of vaccines, especially against viral infections, as some have taken the yearly flu vaccine and ended up with multiple upper respiratory tract infections during that year [19].

Similar to the data from France [20], Australia [21], UK [22], and the US [17], females were more inclined than males not to take the COVID-19 vaccine, probably because females tend to express concerns about the unforeseen effects of vaccines [22] and mistrust the COVID-19 vaccine itself, which makes them fearful and reluctant to take it. Other reasons that might affect women’s willingness to take the vaccine, are discomfort in response to vaccination, feelings toward previous vaccinations, and other factors belonging to personal and physical feelings [23]. However, our sample size is small, and the number of surveyed women was greater than that of men, which could have affected our results.

Our results showed that being married compared to single was significantly associated with lower odds of willing to receive the COVID-19 vaccine, contrary to the findings of a study conducted in China [24]. Part of the reasoning might be that married couples have more protective attitudes and higher adherence to protective behaviors than single people because, besides self-protection, they are responsible for their families. Thus, they tend to think and worry about the vaccine side effects, such as an irreversible illness that could lead to reduced family functionality and their inability to raise their kids.

Our study could not demonstrate a significant association between the willingness to receive the COVID-19 vaccine and the practice score. Our results have shown that people will keep taking precautions against the virus the same way they did before receiving the vaccine; this practice intention would make the living environment relatively safer while reducing stress. The other way round, some might be reluctant to receive the vaccine since precautions will remain the same after vaccination. Additional studies are necessary to elucidate this particular point.

### Public health implications

These findings highlight the importance of public awareness measures to alleviate concerns about vaccine safety and efficacy. Another point derived from our results is that health education and communication from authoritative sources are crucial to breakdown existing barriers to intent to vaccinate, which can be achieved by doing more webinars and explaining to people the importance of vaccine-acquired immunity to encourage them to receive it.

### Limitations

Our study has several limitations. Its cross-sectional design does not allow us to infer causality. We asked individuals about their intent to be vaccinated at a time when vaccines were not yet available; thus, the actual intention to be vaccinated against COVID-19 could be different and, hence, responses may be different when the vaccine becomes available. Our sample might not be representative of the Lebanese population due to its small size. A selection bias is possible due to the snowball technique used to recruit the sample. Despite the rich demographic measures, we could have missed some subgroups of the population and some factors associated with the willingness to receive the vaccine that could have changed our results, predisposing us to a residual confounding bias.

## Conclusion

Overall, our findings revealed a high percentage of people (40%) who strongly disagreed with receiving the vaccine, mainly females, married participants, and those who have a general vaccine hesitancy. Moreover, no significant association was found with knowledge, attitude, or prevention practice regarding COVID-19. Targeted efforts are necessary to increase acceptance of a COVID-19 vaccine among the Lebanese population to control the COVID-19 pandemic. Further studies with a larger sample size are warranted to validate our results and provide better insights into the underlying reasons for refusing vaccination.

## Data Availability

All data generated or analyzed during this study are not publicly available to maintain the privacy of the individuals’ identities. The dataset supporting the conclusions is available upon request to the corresponding author.

## Abbreviation

SPSS: Statistical package for the Social Sciences
